# Febrile neutropenia incidence and the variable toxicity profile between brand and generic docetaxel in the adjuvant treatment of breast cancer with docetaxel and cyclophosphamide regimen

**DOI:** 10.31744/einstein_journal/2023AO0486

**Published:** 2023-12-12

**Authors:** Flávia Viécili Tarcha, Ana Luísa de Castro Baccarin, Lilian Arruda do Rêgo Barros, Erika Bushatsky Andrade de Alencar, Auro del Giglio, Felipe José Silva Melo Cruz

**Affiliations:** 1 Instituto Brasileiro de Controle do Câncer São Paulo SP Brazil Instituto Brasileiro de Controle do Câncer , São Paulo , SP , Brazil .; 2 Centro Universitário FMABC Santo André SP Brazil Centro Universitário FMABC , Santo André , SP , Brazil .

**Keywords:** Breast neoplasms, Febrile neutropenia, Granulocyte colony-stimulating factor, Drugs, generic, Docetaxel, Cyclophosphamide

## Abstract

**Objective:**

To assess the incidence of febrile neutropenia without primary granulocyte colony-stimulating factor prophylaxis in patients undergoing chemotherapy with adjuvant docetaxel and cyclophosphamide, and to evaluate the toxicity profile of brand-name docetaxel (Taxotere ^®^ ) and the generic formulation.

**Methods:**

This retrospective study was conducted using data obtained from electronic medical records of patients treated at a Brazilian cancer center. Patients with breast cancer who underwent adjuvant treatment between January 2016 and June 2019 were selected. Data were analyzed using chi-square and Fisher correlation of variables, and multivariate analyses were adjusted for propensity score.

**Results:**

A total of 231 patients with a mean age of 55.9 years at the time of treatment were included in the study. The majority (93.9%) had luminal histology, 84.8% were at clinical stage I, and 98.2% had a good performance status. The overall incidence of febrile neutropenia in the study population was 13.4% (31 cases). The use of brand-name docetaxel (Taxotere ^®^ ) was the only factor associated with febrile neutropenia occurrence (OR= 3.55, 95%CI= 1.58-7.94, p=0.002).

**Conclusion:**

In patients with breast cancer who require treatment with adjuvant docetaxel and cyclophosphamide regimen, the toxicity profile differs between brand-name and generic docetaxel. Regardless of the formulation used, the incidence of febrile neutropenia was less than 20%, which may allow for the omission of primary prophylactic granulocyte colony-stimulating factor use in this setting.

## Summary

In Brief

Tarcha et al. assessed febrile neutropenia in patients undergoing adjuvant chemotherapy with docetaxel and cyclophosphamide and compared the toxicity of brand-name Taxotere® and generic docetaxel. The incidence of febrile neutropenia was <20%,suggesting the potential omission of prophylactic granulocyte colony-stimulating factor use in this setting.

## Abstract (key points)

Highlights

The overall incidence of febrile neutropenia in the study population was 13.4% (31 cases).

Brand-name docetaxel (Taxotere®) use was the only factor associated with the occurrence of febrile neutropenia.

No statistically significant differences in progression-free survival rates between brand-name and generic docetaxel.

## Graphical abstract

**Figure:**
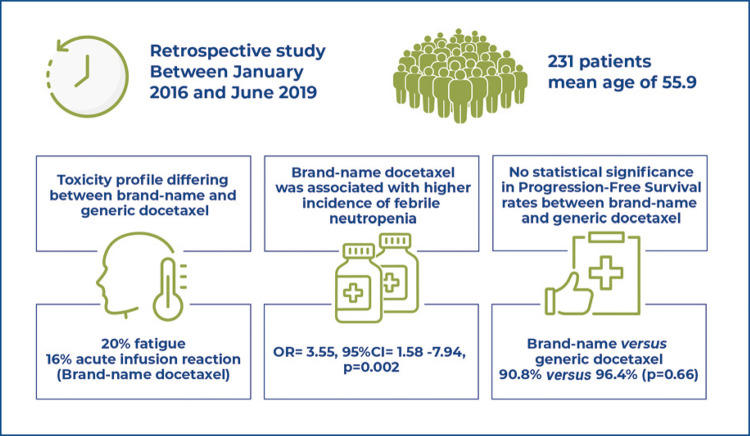

## INTRODUCTION

Despite adequate treatment, breast cancer carries a 4-7% risk of locoregional recurrence and a risk of distant recurrence of up to 30%, depending on the clinical stage. The main objective of adjuvant treatment is to reduce recurrence and increase overall survival in patients with breast cancer. ^( 1 - 3 )^ The decision to use adjuvant treatment depends on pathological features such as tumor size, grade, histological subtype, lymph node involvement, and lymphovascular invasion. ^( 1 - 2 )^ For uncertain cases, personalized tumor genetic analysis tests such as Oncotype DX ^®^ and MammaPrint ^®^ can help determine individual recurrence risk and predict the potential benefit of adjuvant chemotherapy. ^( 1 , 4 , 5 )^

In selecting a treatment regimen, it is essential to consider the tumor’s pathological and clinical characteristics and the patient’s age, performance status, comorbidities, previous treatments, and the possible side effects associated with treatment. ^( 2 , 5 , 6 )^ In an attempt to minimize side effects, taxanes have replaced anthracyclines for patients at intermediate risk (early breast cancer, hormone receptor-positive with an indication for chemotherapy based on pathological risk or assessed via genetic testing). The combination of docetaxel and cyclophosphamide (TC) is superior to the combination of doxorubicin and cyclophosphamide (AC). ^( 7 - 9 )^ With a median follow-up of 7 years, Jones et al. found that the difference in disease-free survival between TC and AC was 81% *versus* 75%, respectively, with a hazard ratio (HR) of 0.74, and overall survival was 87% TC *versus* 82% AC with an HR 0.69. ^( 10 )^

There were notable differences in the toxicity profiles of each regimen. Patients treated with AC experienced higher incidences of nausea, vomiting, and cardiotoxicity. In contrast, febrile neutropenia was the most significant toxicity associated with the TC regimen, with a rate of 5% and even higher in patients over 65 years of age (8%). Notably, no prophylactic granulocyte colony-stimulating factor (G-CSF) was utilized during their study.

Considering the varying incidence rates of febrile neutropenia associated with the docetaxel and cyclophosphamide regimen reported in the literature, which ranges between 5% and 70%, it is unclear whether primary prophylaxis is necessary. The main oncological guidelines recommend the use of G-CSF for prophylaxis when the risk of developing neutropenic fever is greater than or equal to 20%. ^( 11 - 15 )^ When used as primary prophylaxis, the main adverse effect of the drug is bone pain, with a prevalence ranging from 20% to 60%. Other adverse events include fever, leukocytosis, and allergic reactions. ^( 16 - 18 )^ Furthermore, G-CSF is considered a high-cost medication, primarily used for prophylaxis in patients undergoing cancer treatment, and is currently not included in the norms and recommendations of the Brazilian Ministry of Health. This makes the practice difficult in many health centers in the country, especially within the context of the Unified Health System (SUS - *Sistema Único de Saúde* ). ^( 19 )^

To tackle the increasing healthcare costs, the utilization of generic chemotherapeutics has become prevalent in the field of oncology, particularly within the SUS. Approval is granted based on bioequivalence and equivalence tests; nevertheless, bioequivalence values differ significantly among patients, and there is a lack of literature comparing the quality attributes of generic and branded chemotherapy formulations and their adverse effects. ^( 20 )^

This study aimed to investigate the occurrence of febrile neutropenia in breast cancer patients who received adjuvant combination therapy (CT) without G-CSF prophylaxis at a Brazilian oncology center serving patients with both public and private health insurance. Additionally, we sought to analyze the frequency of febrile neutropenia and the variable toxicity profile between the branded and generic formulations of Taxotere ^®^ used in this treatment approach.

## OBJECTIVE

To assess the incidence of febrile neutropenia without primary G-CSF prophylaxis in patients undergoing chemotherapy with adjuvant docetaxel and cyclophosphamide, and to evaluate the toxicity profile of brand-name docetaxel (Taxotere ^®^ ) and the generic formulation.

## METHODS

### Study design

We retrospectively analyzed medical records from electronic databases of the PR systems and Tasy programs, which are electronic medical record systems, to identify patients who had undergone adjuvant treatment with TC for breast cancer. The requirement for informed consent was waived because of the study’s retrospective design.

The study was approved by the research ethics committee of the *Instituto Brasileiro de Controle do Câncer* (CAAE: 28427420.2.0000.0072; # 3.852.273).

### Patient selection

We reviewed the medical records of 293 patients who were diagnosed with breast cancer and indicated for adjuvant chemotherapy with docetaxel and cyclophosphamide after surgery at the the *Instituto Brasileiro de Controle do Câncer-São Camilo Oncology hospital* .

Patients older than 18 years who had received at least one cycle of TC between January 2016 and June 2019 were included, while patients with metastatic breast cancer (44 patients) or those who had received G-CSF as primary prophylaxis (18 patients) were excluded from the study.

Of the 293 patients identified, we selected and analyzed 231 medical records. The extracted data included age at the onset of treatment, Eastern Cooperative Oncology Group (ECOG) performance scale, clinical-pathological stage of the tumor, immunohistochemistry, body mass index (BMI), use of previous chemotherapy, previous radiotherapy, incidence of febrile neutropenia, infusion reactions to docetaxel, incidence of acute toxicities, and type of docetaxel used (generic and Taxotere ^®^ ).

All patients received ondansetron and dexamethasone as premedication, and the only difference between chemotherapy protocols was the administration of either Taxotere ^®^ or generic docetaxel based on their health insurance coverage. The chemotherapy treatment was identical for all patients who were administered the generic formulation. Toxicities were classified into grades based on the typology proposed in the Common Terminology Criteria for Adverse Events (CTCAE) version 4.0.

### Statistical analysis

Patient characteristics were reported using their distributions and percentages. The χ ^2^ test was used to evaluate categorical variables, and the *t* -test was used to evaluate continuous variables. Multivariate analyses were performed by associating febrile neutropenia and the brand of docetaxel, adjusted for propensity score (age, BMI, ECOG, previous chemotherapy, and previous radiotherapy were included in the model). We evaluated progression-free survival (PFS), and the log-rank test was used to assess whether there was a difference in PFS based on brand or generic docetaxel. Statistical significance was set at p<0.05, and the analyses were conducted using Stata17.0 ^®^ (STATA Inc., Texas, USA).

## ❚RESULTS

### Patient population

All 231 patients in the study were female, with a mean age of 55.9 years. Of these, 84.8% presented with initial stage I, 93.9% had luminal tumors, and the vast majority, 98.2%, had good performance status (ECOG 0). Overall, 68.8% (159) of patients were treated with generic docetaxel ( Table 1 ). Regarding treatment for previous neoplasms, 11.3% had already been exposed to radiotherapy, and 9.5% of the patients had received prior chemotherapy, 20 of whom had undergone treatment with an anthracycline.

**Table 1:** Descriptive analysis of variables

Variables	
Age at treatment (years)	
Mean	55.9
Median (Min;Max)	55 (35; 79)
Age (years), n (%)	
<65	180 (77.9)
≥65	51 (22.1)
BMI, n (%)	
Normal	58 (25.1)
Overweight	89 (38.5)
Obesity	63 (27.3)
Severe obesity	21 (9.1)
IHC, n (%)	
Luminal	217 (93.9)
Triple negative	14 (6.1)
Stage, n (%)	
I	196 (84.8)
II	32 (13.8)
III	3 (1.3)
ECOG, n (%)	
0	227 (98.2)
1 & 2	4 (1.8)
Prior CT, n (%)	
No	209 (90.5)
Yes	22 (9.5)
Previous RT, n (%)	
No	205 (88.7)
Yes	26 (11.3)
Docetaxel, n (%)	
Generic	159 (68.8)
Taxotere ^®^	72 (31.2)

BMI: body mass index; IHC: immunohistochemistry; ECOG: Eastern Cooperative Oncologic Group; CT: chemotherapy; RT: radiotherapy.

### Toxicities

Chemotherapy was suspended in 17 patients; four discontinued treatment owing to febrile neutropenia and three because of regimen-related toxicities such as mucositis, neuropathy, and hand-foot syndrome. Other causes of treatment discontinuation include patient preference, acute myocardial infarction, and severe allergic/infusion reactions (AIRs) to docetaxel. The incidence of infusion reaction to docetaxel was 24.6% in the general population, and among the 57 patients who manifested signs of allergy, 21 experienced a recurrence at least once. The use of branded docetaxel exhibited a greater incidence of RIA compared to generic medications (16% *versus* 4.3%, p<0.01).

The general population experienced the highest rates of toxic effects for symptoms such as nausea, fatigue, and diarrhea, with percentages of 32%, 30.7%, and 23.4%, respectively. However, these symptoms were effectively managed and well-tolerated, and no grade 4 toxicities were reported. An analysis of various toxic effects, including nausea, vomiting, diarrhea, constipation, mucositis, myalgia, neuropathy, and hand-foot syndrome, did not reveal any correlation with the specific type of docetaxel used. However, the use of Taxotere ^®^ was significantly associated with the occurrence of fatigue and acute infusion reaction, with percentages of 20% and 16%, respectively ( Table 2 ).

**Table 2:** Toxicity analysis in the general population, population receiving generic docetaxel and receiving Taxotere ®

General toxicities	Total n (%)	Generic drug n (%)	p value	Drug brand n (%)
Nausea				
Grade 1 or 2	74 (32)	53 (22.9)	0.41	20 (8.6)
Grade 3	0 (0.00)	0 (0.00)		0 (0.00)
Vomit				
Grade 1 or 2	17 (7.3)	13 (5.6)	0.62	7 (3.0)
Grade 3	0 (0.00)	0 (0.00)		0 (0.00)
Fatigue				
Grade 1 or 2	71 (30.7)	24 (10.3)	<0.01	47 (20.3)
Grade 3	1 (0.43)	1 (0.43)		0 (0.00)
Diarrhea				
Grade 1 or 2	54 (23.4)	36 (15.5)	0.65	18 (7.7)
Grade 3	1 (0.43)	1 (0.00)		0 (0.00)
Constipation				
Grade 1 or 2	11 (4.7)	6 (2.5)	0.32	5 (2.1)
Grade 3	0 (0.00)	0 (0.00)		0 (0.00)
Mucositis				
Grade 1 or 2	30 (13)	20 (8.6)	0.78	10 (4.3)
Grade 3	1 (0.43)	0 (0.00)		0 (1.3)
Myalgia				
Grade 1 or 2	19 (8.2)	16 (6.9)	0.19	3 (1.2)
Grade 3	1 (0.43)	0 (0.00)		1 (0.43)
Neuropathy				
Grade 1 or 2	7 (3.03)	4 (1.7)	0.68	3 (1.2)
Grade 3	0 (0.00)	1 (0.43)		0 (0.00)
HFS				
Grade 1 or 2	1 (0.43)	1 (0.43)	1.00	0 (0.00)
Grade 3	0 (0.00)	0 (0.00)		1 (0.43)
AIR				
Present	57 (24.6)	20 (4.3)	<0.01	37 (16.0)
Absent	174 (75.4)	139 (60.1)		35 (15.1)

HFS: hand foot syndrome; AIR: acute infusion reaction.

### Febrile neutropenia

The overall incidence rate of febrile neutropenia in the population was 13.4%. The incidence rate was 15.5% among individuals who experienced febrile and grade 4 neutropenia. Among patients who developed febrile or grade 4 neutropenia after the first cycle, 22 were hospitalized, and 25 underwent secondary prophylaxis with colony-stimulating factors; however, two had a new episode of FN. The mean number of days of hospitalization was five, with a minimum of 1 and a maximum of 11 days. When comparing the incidence of febrile neutropenia and the combined outcome of febrile and Grade 4 neutropenia according to docetaxel type, the rate was slightly higher in the brand name Taxotere ^®^ population compared to the generic drug: 7.4% *versus* 6.0% (p=0.002) and 8.2% *versus* 7.3% (p=0.002), respectively ( Table 3 ). Multivariate analysis of the association of febrile neutropenia and type of chemotherapy (generic *versus* Taxotere ^®^ ) was adjusted for propensity score, and the model included the main characteristics considered relevant for the occurrence of the event: age, BMI, ECOG, previous chemotherapy, and previous radiotherapy. The type of chemotherapy was the only one associated with the incidence of febrile neutropenia (p<0.01).

**Table 3:** Incidence of febrile neutropenia (primary endpoint) and incidence of febrile neutropenia + neutropenia grade 4 (secondary endpoint)

Outcome	Total n (%)	Generic drug n=159 n (%)	Taxotere ^®^ n=72 n (%)	p value
Febrile neutropenia				
Yes	31 (13.4)	14 (6.0)	17 (7.4)	0.002
No	200 (86.6)	145 (62.9)	55 (23.8)	
Neutropenia grade 4 + febrile neutropenia				
Yes	36 (15.5)	17 (7.3)	19 (8.2)	0.002
No	195 (84.5)	142 (61.5)	53 (23)	

### Progression-free survival

The analysis of PFS was conducted 3 years after the initial assessment. The overall population exhibited a 5-year PFS of 92.4%, 95%CI= 85.7-96.0%. Subgroup analysis showed that the PFS rates for the brand and generic groups were 90.8% (95%CI= 81.8-95.5%) and 96.4% (95%CI= 96.3-99.1%), respectively (p=0.66).

## DISCUSSION

In this retrospective analysis conducted at a Brazilian oncology center, we demonstrate that the use of G-CSF as primary prophylaxis can be omitted in breast cancer patients treated with adjuvant TC regimen. According to the recommendations of major oncology guidelines, including the American Society of Clinical Oncology (ASCO) and the European Society for Medical Oncology (ESMO), G-CSF is indicated as primary prophylaxis when the risk of febrile neutropenia secondary to a chemotherapy regimen is 20%. ^( 7 , 21 )^

Granulocyte colony-stimulating factor is an effective medication for reducing the incidence of febrile neutropenia and treating it when it occurs. Despite its benefits, its use remains challenging in public health in Brazil owing to increased treatment costs, accessibility for administration, and potential side effects such as bone pain, fever, leukocytosis, and allergic reactions. ^( 22 - 24 )^ Febrile neutropenia is the most frequent toxicity associated with the TC regimen; however, its reported incidences in the literature vary widely, depending on the studied population.

We found a lower incidence of febrile neutropenia compared to that reported in an analysis of two other Brazilian centers when patients did not undergo primary prophylaxis (13.4% *versus* 24%, respectively). It should be noted that Gagliato et al. ^( 1 )^ were unable to determine a subgroup of patients at higher risk. Factors related to patient characteristics, such as age, have been associated with risk factors for febrile neutropenia. The USO-9735 study reported that the incidence of febrile neutropenia in patients over 65 years of age was 8%, which was twice as high as the incidence observed in younger patients (4%). Other previously identified risk factors include prior chemotherapy or radiation therapy, poor performance status, presence of comorbidities, and advanced disease. ^( 25 , 26 )^

Despite the febrile neutropenia rate reported in the general population of this study, we identified a statistically significant factor associated with the occurrence of febrile neutropenia: the use of brand-name docetaxel (Taxotere ^®^ ). Additionally, patients who used the original drug also experienced higher rates of acute infusion reactions to docetaxel compared to the generic drug.

The use of generic drugs is a common practice in health care because of the expectation of cost reduction. However, the release of these drugs requires that they be equivalent or bioequivalent to the original formulations, allowing for some differences in active ingredients. Some studies have evaluated the differences in toxicities between generic and original formulations. ^( 27 - 30 )^

In 2008, 31 generic docetaxel formulations from 14 countries were analyzed in comparison to Taxotere ^®^ . The results showed that 90% of generic products did not have similar characteristics to the original product, potentially containing insufficient amounts of active drug, high levels of impurities, or both. ^( 31 )^ Garrido-Siles et al. reported varying incidences of acute infusion reactions and skin reactions at a Spanish center when they compared four distinct presentations of docetaxel. Thus, the presence of impurities and discrepant characteristics between generic and original formulations may influence the occurrence of different toxicities. ^( 32 )^

Although we observed varying adverse effects between docetaxel formulations and a higher incidence rate of febrile neutropenia with Taxotere ^®^ , there was no statistically significant difference in the 5-year PFS when comparing the two drugs. The low number of events and small sample size reduced the power to detect any difference in PFS, although there were numerically more progressions in the generic drug group (13 *versus* 5).

The study has certain limitations, including the retrospective design, which relies on electronic databases completed by various oncologists. Despite efforts to ensure data accuracy, the possibility of incomplete or missing information cannot be entirely ruled out. Additionally, as a single-center study, the results may not be generalizable to other populations or healthcare settings. Nevertheless, this study provides important insights into the use of generic docetaxel in adjuvant chemotherapy for breast cancer, particularly regarding the incidence of febrile neutropenia and acute toxicities. These findings provide valuable insights for healthcare professionals involved in the treatment of patients with cancer and may help inform decision-making regarding the use of prophylactic measures and the management of chemotherapy-related toxicities.

## CONCLUSION

Based on the present study, the incidence of febrile neutropenia was lower. Consequently, our analysis does not support the use of granulocyte colony-stimulating factor as primary prophylaxis for all patients undergoing the adjuvant regimen with docetaxel and cyclophosphamide. Instead, individualized approaches should be considered. It is important to note that using generic formulations of docetaxel may result in a different toxicity profile compared to the original medication. Therefore, careful monitoring and management of toxicities are necessary depending on the specific medication administered.
